# Associations between Constipation and Use of Levodopa with Nutritional Status, Polypharmacy, and Stage of Parkinson’s Disease

**DOI:** 10.3390/nu16183092

**Published:** 2024-09-13

**Authors:** Paula García-Milla, Samuel Duran-Agüero, Gema Nieto

**Affiliations:** 1 Department of Food Technology, Nutrition and Food Science, Veterinary Faculty, University of Murcia, Regional Campus of International Excellence “Campus Mare Nostrum”, Campus de Espinardo, Espinardo, 30100 Murcia, Spain; ppaulagm@gmail.com; 2 Carrera Nutrición y Dietética, Facultad de Ciencias de la Salud, Universidad Autónoma de Chile, Providencia 7500975, Chile; 3 Escuela de Nutrición y Dietética, Facultad de Ciencias para el Cuidado de la Salud, Universidad San Sebastián, Los Leones, Providencia 7510157, Chile; samuel.duran@uss.cl

**Keywords:** Parkinson’s disease, constipation, nutritional status, levodopa, polypharmacy

## Abstract

Introduction: Parkinson’s disease (PD) is a highly prevalent disease characterized by motor and non-motor symptoms; the latter include constipation, which is considered a prodromal symptom. On the other hand, sarcopenia, polypharmacy, and malnutrition due to deficits are common in PD and lead to poorer health and quality of life. Objective: to associate constipation and use of levodopa with nutritional status, sarcopenia, duration and stage of the disease, and polypharmacy in individuals with PD. Materials and methods: analytical cross-sectional observational study where an online survey was applied to 161 people suffering from PD. Results: a significant association is observed between constipation and BMI (*p* = 0.022), as well as between the use of levodopa with BMI (*p* = 0.049) and polypharmacy (*p* = 0.046). On the other hand, there is a relationship between the average time of PD diagnosis and constipation (*p* = 0.0047). Finally, there is a relationship between SARC-F score applied to those over 60 years of age (*p* = 0.0446) and the use of levodopa. Having sarcopenia, being overweight, and having had the disease for less than five years is associated with a higher probability of experiencing constipation, according to the logistic regression analysis (*p* > 0.005). Conclusion: nutritional assessment and subsequent follow-up is of vital importance to avoid complications that could be associated with levodopa use, constipation, and sarcopenia.

## 1. Introduction

Parkinson’s disease (PD) is one of the most prevalent neurodegenerative diseases after Alzheimer’s disease and chronically and progressively affects the nervous system [1].

According to data provided by the World Health Organization (WHO), the prevalence of PD has doubled in the last 25 years, reaching 8.5 million people with the disease by 2019 [2]. Additionally, about 120,000–150,000 people suffer from the disease in Spain, and 10,000 new cases are diagnosed each year [3]. Meanwhile, in the United States, 90,000 people are diagnosed with the disease every year [4]. Chile has a high prevalence of PD compared to the rest of Latin America, with its prevalence increasing by approximately 19.9% in the period 1990–2016. In 2018, the prevalence of PD in Chile was 160.7/100,000 inhabitants, with a men/women ratio of 1.03 [5]. Moreover, it is among the top five Latin American countries with increased mortality due to PD [6]. This mortality has been on the rise since 1990, with an increase in the adjusted mortality rate from 0.94 to 2.0 per 100,000 inhabitants in the period 1990–2009. In addition, the greatest increase in mortality in Chile occurred in the period 1999–2002, with a rise of 47.8% [7].

Although the disease does not occur exclusively in old age, 70% of those affected are over the age of 65, while regarding its early onset, that is, in the 30–50 age group, it accounts for only 10% of patients. Juvenile onset, which begins at 21 years of age, affects 0.25% of individuals [8]. Consequently, age is the most important risk factor for the development of the disease; therefore, the prevalence of PD is expected to increase dramatically over the next years in view of population aging [9].

Among the symptoms of PD, constipation is a highly prevalent non-motor feature; between 50% and 80% of patients suffer from this condition, which significantly affects the quality of life and health of individuals with PD [10,11,12]. According to published studies, constipation could be a predictor of Parkinson’s disease (PD), becoming a prodromal symptom of PD, as it may appear several years before the onset of motor symptoms and diagnosis of the disease. Additionally, it has been correlated with the duration and severity of the disease [12,13,14,15,16].

On the other hand, weight loss and malnutrition are common problems in PD that lead to poorer health and quality of life [17]. Evidence has shown that people suffering from this disease are more likely to experience a 3–5% weight loss, which is associated with greater disability and disease severity in the long term [18]. In addition, there would be a dose–response association between body mass index (BMI) at diagnosis and mortality; therefore, the assessment of nutritional status and weight changes is essential [19].

On the other hand, the prevalence of sarcopenia in older people ranges from 10% to 27% [20,21]. Although available data are sparse in Latin America, a prevalence of 19.1% has been reported in Chile, while in Costa Rica it reaches 10.26% [21].

Furthermore, it is important to note that sarcopenia is highly prevalent in individuals with PD, being three times higher than in people not affected by the disease [22], ranging from 10.9% to 31.4% [23] and co-occurring with PD. According to some researchers, sarcopenia could be related not only to the disease itself but also to the use of levodopa in these patients [24].

Given the relevance of knowing the constipation situation and the nutritional status, the objective of this study is to associate constipation and the use of levodopa with nutritional status, sarcopenia, duration and stage of the disease, and polypharmacy in people with PD.

## 2. Materials and Methods

An analytical cross-sectional observational study was conducted from 12 October 2023 to 15 January 2024. Participants consisted of adults and older people with PD, with or without the assistance of their caregivers at the moment of completing the survey. Exclusion criteria included people with dementia or those who were not able to answer the questions due to cognitive impairment. The inclusion criteria were any person over 18 years of age with Parkinson’s disease who could complete the survey, either independently or with the help of their caregivers.

Due to the nature of the study and the target population, it was decided to conduct the data collection using causal or random sampling (non-probabilistic), since there is no official data on the prevalence of Parkinson’s disease in Chile and Latin America [25].

### 2.1. Survey

The instrument was constructed and subsequently validated by a group of nutritionists, experts in the field of food and nutrition, who reviewed the relevance of each question according to the research objective. Then, a small sampling including five patients with PD was conducted in order to assess the instrument’s understandability. Once the survey was approved, it was disseminated via Google Forms using a non-probabilistic sampling method.

The survey was spread through social networks such as Instagram and especially Facebook, where it was shared on a weekly basis in groups of patients with PD. In order to avoid survey duplication, participants were asked to create a code consisting of the initials of their first and last name followed by the first 4 digits of their ID number. In this way, the survey was identified at the time of analysis, but at the same time, the anonymization ensured compliance with the protection of participants’ data.

The survey was developed including questions categorized by the following components: personal history of Parkinson’s disease, assessment of nutritional status, constipation, and polypharmacy.

Most of the questions were designed as multiple-choice items. Open-ended questions were labeled and coded to favor data analysis.

It is important to highlight that internet usage was not considered a barrier, as official data from the Chilean government’s Undersecretary of Telecommunications indicate that 93% of households report having their own paid internet connection [26], with a speed that places the country among the top in the world [27]. Additionally, 71% of the population in Latin America were internet users by 2022, with Haiti being the country with the lowest connection, with only 50% of its inhabitants online. It is worth noting that Chile has the highest social media penetration in households [28].

On the other hand, although people over the age of 60 have an internet usage rate of 44% compared to 83.4% for those under 59 years [29], this survey allowed respondents to be assisted or have their caregivers respond on their behalf, ensuring that each answer was in relation to the person with Parkinson’s disease. Furthermore, this study included individuals aged 18 and over and was not exclusive to older adults.

#### 2.1.1. Assessment of Nutritional Status

In subjects over 60 years of age, nutritional status was assessed according to the Mini Nutritional Assessment (MNA) screening tool, whose score ranges from 0 to 14 points, where 0–7 points: malnutrition; 8–11 points: at risk of malnutrition, and finally 12–14 points: normal nutritional status [30,31].

In addition, nutritional status was assessed according to body mass index (BMI), where information on weight and height was reported by the participants.

For the classification of nutritional status according to BMI, researchers used age-adjusted scores. In the case of individuals aged 18–59 years (adult population), a BMI of <18.5 kg/mt^2^ was considered underweight; 18.5 to 24.9 kg/mt^2^ normal weight; 25 to 29.9 kg/mt^2^ overweight; >30 kg/mt^2^ obesity [32]. With respect to individuals over 60 years of age (older people), <22 kg/mt^2^ was considered underweight; 22 to 26.9 kg/mt^2^ normal weight; 27 to 29.9 kg/mt^2^ overweight; and >30 kg/mt^2^ obesity [33].

#### 2.1.2. Assessment of Sarcopenia

Since the data collection was conducted through an online survey, which made it impossible to take anthropometric measurements, it was decided to assess the risk of sarcopenia using the screening tool or questionnaire for sarcopenia detection, also known as the SARC-F.

The SARC-F questionnaire consists of five questions with a score ranging from 0 to 2 points per item. A final score greater than 4 points is indicative of sarcopenia [34]. Data analyzed for the sarcopenia score were obtained from the group of people aged over 60 years, which corresponds to the definition of older people in whom the SARC-F questionnaire has been validated.

#### 2.1.3. Assessment of Constipation

Constipation is defined as a decrease in the frequency of bowel movements, accompanied by an increase in stool consistency, as well as difficulty in passing stools [35,36]. Based on this definition, respondents were asked to classify their bowel movements according to their consistency by showing them the Bristol Stool Chart along with a description of each stool type. Types 1 and 2 stools are indicative of constipation; types 3 and 4 are normal feces; type 5 corresponds to pre-diarrhea; and finally, types 6 and 7 are diarrheal stools [37]. For data-analysis purposes, the latter (types 5, 6, and 7) were grouped together.

In addition, participants were asked about the frequency of going to the toilet and were given the following alternatives: once or less per week, twice a week, every other day, and daily.

Additionally, participants were asked if they suffered from or had been diagnosed with constipation (Yes or No), as well as whether they were taking any supplements or medications to manage constipation, specifying which ones they were taking.

#### 2.1.4. Assessment of Parkinson’s Disease and Polypharmacy

Two questions were used to evaluate the overall situation of the disease; the first consisted of an open-ended question: How long has it been since the diagnosis of the disease? and the second was a multiple-choice question: What stage of Hoehn and Yahr are you in? A description of each stage (ranging from 1 to 5) was provided in order to ease understanding.

For the statistical data analysis, stages 4 and 5 were grouped together. Finally, respondents were asked about the use of levodopa (the gold-standard medication for the control of PD symptoms), and they could answer Yes or No.

The assessment of polypharmacy was based on the question: do you take more than three medications? [38], where the possible answers were Yes or No.

### 2.2. Ethics

The study was based on the Declaration of Helsinki regarding work involving human beings and agreed with the Singapore Statement on Research Integrity. In addition, it was approved by the Ethics Committee of the University of Murcia, Spain, which gave a favorable report (ID: 4729/2023). Each participant read the informed consent and, if they agreed to participate, the online survey was displayed.

### 2.3. Statistics

Statistical analysis was performed using the statistical package STATA v16. Descriptive statistics were used to assess general data such as age, weight, height, body mass index (BMI), SARC-F and Mini Nutritional Assessment screening score, and years since diagnosis with Parkinson’s disease.

Fisher’s exact test was used to evaluate associations, and multiple comparisons were tested using the ANOVA test according to Bonferroni for comparison analyses between constipation and the variables age, SARC-F screening, and years with the disease, as well as comparisons in the use of levodopa and the variables SARC-F screening, age, and years with Parkinson’s disease.

Additionally, a logistic regression was performed to evaluate the risk of constipation according to the variables of the SARC-F questionnaire, years with Parkinson’s disease, Hoehn and Yahr stages, use of medications or supplements for constipation, levodopa consumption, and nutritional status according to BMI and MNA.

A *p*-value of <0.05 was considered statistically significant for all the analyses.

## 3. Results

**Figure 1:**
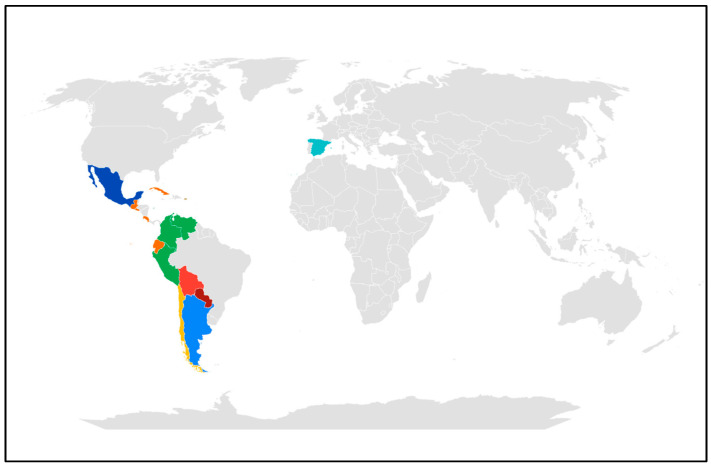
Country of origin.

A total of 161 participants with PD completed the survey; 66.5% were female, and 33.5% were male. In relation to the country of origin, the study involved 15 nations, and individuals from Mexico accounted for the largest number of participants, followed by people from Chile and Spain (Figure 1). Table 1 describes the sample in terms of age, weight, height, BMI, MNA score, and SARC-F score. It shows that the average age of the sample was 60.8 ± 12.1 years, the minimum age was 21 years, and the maximum age was 87 years. The BMI score was 26.4 ± 5.69; thus, it can be inferred that average nutritional status was normal according to the BMI. However, when individuals over 60 years of age were evaluated using the MNA screening tool, the assessment yielded an average score of 10 ± 3.24, which falls into the category of nutritional status at risk of malnutrition due to deficit, with 37% of the respondents having a score indicative of being at risk of malnutrition (Table 2). Additionally, participants were asked about the number of years since they had been diagnosed with Parkinson’s disease; the average corresponded to 9.41 ± 6.97 years with a minimum of less than 1 year and a maximum of 40 years of evolution (Table 1).

Assessment of constipation was conducted by asking the participants to report whether or not they had constipation. Subsequently, consistency and frequency of stools were classified according to the Bristol scale; 72% of the subjects reported having constipation; however, 81% stated that their stool consistency corresponded to type 3 and 4 (normal), followed by 64% who identified their stools as type 1 and 2 (constipation). When examining toileting frequency, 54% indicated a daily frequency, followed by 48% who mentioned going to the toilet every other day, and finally, 41% reported a frequency of twice a week, and 18% once or less per week. When asked about the use of medications or supplements for managing constipation, 52.5% of the sample reported not using any medication or supplement. Then, with a much lower percentage, 17.50% reported using some type of osmotic laxative, followed by 16.2% who reported using natural supplements or fiber-rich foods, and finally, 13.7% reported using stimulant laxatives (Table 2).

Regarding the description of the sample in relation to PD, 87.5% reported levodopa use. In addition, when asked in which stage of the disease the person was, 30.4% indicated being in Stage 1, followed by 26.7% in Stage 2, 22.3% in Stage 3, and 20.5% in Stage 4 and Stage 5, according to the Hoehn and Yahr scale (Table 2).

Table 3 shows the results of the association between constipation and the variables sex, nutritional status, stage of the disease, use of levodopa, and polypharmacy. The test showed a statistically significant association between constipation, as assessed by the Bristol scale, and BMI (*p* = 0.022). However, findings were not the same when the evaluation was conducted according to nutritional status as assessed by the MNA screening (*p* = 0.825). With respect to the other variables, there is no significant association between constipation and sex, stage of the disease, use of levodopa, or polypharmacy. On the other hand, when assessing the association between the use of levodopa and the variables nutritional status, stage of the disease, sex, and polypharmacy, we found that there is a statistically significant association between the use of levodopa and BMI (*p* = 0.049), as well as between the use of levodopa and the respondents’ polypharmacy (*p* = 0.046) (Table 4).

The multiple comparison test according to constipation as assessed by the Bristol scale with age, sarcopenia (assessed by SARC-F), and number of years with the disease demonstrated that there is a statistically significant difference in the average time of PD diagnosis according to constipation (*p* = 0.0047). The average time of diagnosis for Parkinson’s disease was significantly greater in Stages 1 and 2 compared to Stages 3 and 4 (*p* = 0.004). However, there was no statistically significant difference in the average time of diagnosis for PD between Stage 1 and Stage 2 compared to Stages 5, 6, and 7 (*p* = 0.236), nor was there a significant difference in the average time of diagnosis for PD between Stage 3 and Stage 4 compared to Stages 5, 6, and 7 (*p* = 1.000) (Table 5).

Finally, multiple comparisons evaluating levodopa use and sarcopenia (assessed by SARC-F score), age, and time suffering from Parkinson’s disease showed a statistically significant difference with the SARC-F score applied to individuals over 60 years of age (*p* = 0.0446) (Table 6), establishing a relationship between the use of the aforementioned medication and sarcopenia.

A logistic regression model was performed for the response variable of constipation. This analysis included independent variables such as Hoehn and Yahr stages, nutritional status according to BMI and MNA, years with Parkinson’s disease, levodopa consumption, and use of medications for constipation.

According to the results, the model for the probability of constipation based on BMI (Table 7) shows a good fit (*p* = 0.5409). It can be observed that patients with a positive sarcopenia questionnaire (score > 4) have a 2.9 times higher probability of having constipation compared to patients without sarcopenia, according to the SARC-F questionnaire (*p* = 0.036).

Additionally, the duration of Parkinson’s disease, which was categorized into less than 5 years and between 5 and 10 years, indicates that patients with a diagnosis of 5 years or less have a 3.7 times higher probability of having constipation compared to patients with a diagnosis between 5 and 10 years (*p* = 0.026).

When analyzing the body mass index (BMI) of the respondents, patients with overweight BMI scores have a 4.5 times higher probability of having constipation compared to patients with underweight BMI scores (*p* = 0.028).

Finally, there is a trend in the use of stimulant laxatives, suggesting that individuals who use this type of medication might have a protective effect against constipation, which is expected given the primary function of these medications.

Table 8 shows the probability of constipation according to nutritional status based on MNA. It shows a good fit with a *p*-value of 0.5021. In this model, only two variables were statistically significant. It is observed that respondents with sarcopenia have a 2.8 times higher probability of having constipation compared to patients without sarcopenia (*p* = 0.033), while respondents with a diagnosis of 5 years or less with Parkinson’s disease have a 3.5 times higher probability of having constipation compared to patients with a diagnosis between 5 and 10 years (*p* = 0.021).

## 4. Discussion

The main result of the present study was that people with Parkinson’s disease for less than 5 years have a higher probability of experiencing constipation than those who have had the disease for 5 or more years.

It is important to highlight, as mentioned in the introduction of this work, that constipation is a symptom that could present even before the onset of motor symptoms and diagnosis of the disease, being associated with a higher risk of Parkinson’s disease (PD) or, according to some authors, as a risk factor for PD. Gastrointestinal dysfunction is highly prevalent and directly contributes to increased morbidity, even affecting the clinical management of PD. Constipation in particular can occur in 7% to 70% of cases and is characterized by having fewer than three bowel movements per week. Our results show that 37.2% of the respondents report going to the bathroom two or fewer times a week [39].

On the other hand, the same applies to being overweight and having a positive SARC-F score for sarcopenia, as the analysis shows that people with overweight BMI scores and sarcopenia have a higher probability of experiencing constipation.

Studies that associate the variables examined in our research, especially in people with Parkinson’s disease, are scarce. However, we would like to mention a study in which sarcopenia and its association with constipation in older adults were evaluated, finding a positive association between the two variables (constipation and sarcopenia) (*p* < 0.001) [40].

PD is a disorder that affects men 1.5 to 2 times more than women, and the features of the disease are different between genders. Symptoms such as depression, fatigue, and pain are more prevalent in females, whereas speech problems, rigidity, and sexual dysfunction are more common in men [41]. It should be noted that our study used a virtual survey, which was probably seen mostly by women or caregivers; this might explain the difference between the number of men (34%) and women (66%) who completed the survey.

The average age in our study was 60 years, which is consistent with the age of greatest prevalence and onset of PD [42]; although this disease does not exclusively affect older people, the greatest prevalence is seen in this age group [43].

Our findings show that 72% of respondents reported having constipation; similar results were observed in the study by Ueki A. et al., 2004, which evaluated the association between water intake and constipation, where 71.1% of sampled patients reported constipation [42]. Moreover, in the same study, the authors obtained a mean bowel frequency of once per 3.3 ± 1.1 days, while 25.4% of our respondents reported a frequency of twice a week, with most subjects indicating a daily frequency, with 33.5% [44].

With respect to nutritional status, people suffering from Parkinson’s disease tend to experience nutritional problems, as confirmed by evidence such as a systematic review of 49 clinical studies that included 5.613 subjects, where 23.9% were at risk of malnutrition (RMN) due to deficit according to the MNA. In addition, these researchers found that despite the diagnosis of RMN according to the MNA, the BMI classification was completely different, with most patients being classified as overweight or obese [17]. These results differ greatly (in terms of values) from those found in our study, where, although the assessment using the MNA also showed RMN, these results corresponded to 41.11% of the sample, whereas according to the classification of nutritional status by BMI, most of the subjects had a normal weight with 35.7%, followed by obesity with 23.3%, and finally, overweight and underweight with 20.7% and 20.1%, respectively.

In relation to sarcopenia, this is a disease that is highly prevalent in older people and even more so for those suffering from a disorder such as Parkinson’s disease. According to data found in a systematic review that examined 14 studies, the prevalence of sarcopenia in PD ranged from 10.9% to 31.4%, being associated with the severity of the disease [23]. However, these values differ from those found in another systematic review and meta-analysis, which evaluated 10 studies and determined that the prevalence of sarcopenia ranged from 6% to 55.5% in this population [45]. Despite the results, both studies concluded that sarcopenia is highly prevalent in individuals with PD, and this is also reflected in our findings, where 63.3% of the respondents obtained a SARC-F score greater than 4 points, which is predictive of sarcopenia. According to a study that included 60 participants with PD, it was found that 30% screened positive for sarcopenia when assessed using the SARC-F questionnaire [46], with values below those found in our study. However, de Luna et al. (2023) found values similar to ours, with a positive screening for sarcopenia of 50.8% [24].

It should be noted that both constipation and sarcopenia significantly affect the quality of life and health of people with PD, leading to functional decline and loss of autonomy, among other complications such as fecal impaction, which often requires hospital management [47,48,49].

According to a cross-sectional cohort study that included 1.278 participants which evaluated the association between constipation and sarcopenia, it was found that the prevalence of constipation was associated with sarcopenia and slower gait speed (*p* < 0.05), further finding that participants with constipation had a higher burden of cognitive impairment, disability, and lower quality of life scores [40]. Despite this, our results do not reflect the published findings since we did not find a statistically significant difference between SARC-F and constipation. However, we have observed a statistically significant relationship between constipation and the number of years with PD (*p* = 0.0047). In addition, we found that the average time of diagnosis for PD is significantly longer in stages 1 and 2 of the Bristol scale compared to stages 3 and 4 (*p* < 0.01).

On the other hand, our results have shown a statistically significant association between levodopa use and nutritional status assessed by BMI, as well as with the use of three or more medications (polypharmacy). These results are in line with the findings by Kim Sr. et al. (2016), whose study evaluated 102 patients with PD, finding a relationship between the Hoehn and Yahr stages, duration of levodopa therapy, body weight, and BMI [50].

According to a recent study that assessed the quality of diet in people with Parkinson’s disease (PD), it concluded that the diet is inadequate and of very low quality, which impacts the quality of life and health of individuals, affecting motor symptoms [51]. On the other hand, according to Baert F. et al. [52], dietary intake is crucial for detecting nutritional deficiencies of macro and micronutrients. However, researchers have evaluated dietary fiber intake, yielding results of 26.2 ± 7.7 g/day, while vitamin D and iron intake was 55.9% and 76.5%, respectively [52].

Assessing fiber intake in people with PD is a goal we have set and will undertake in the near future, as in Chile, the fiber intake in the general population is approximately 12.8 g per day [53]. On the other hand, a recent study conducted in an adult population also showed that fiber intake was 12.5 g per day [54]. Both results are well below the recommended intake for fiber. Based on the available scientific evidence, the Food and Agriculture Organization (FAO), the World Health Organization (WHO), and the European Food Safety Authority (EFSA) have established a recommended intake of 25 g per day for adults and indicated that this recommendation could be achieved through the consumption of food sources such as whole grains, legumes, fruits, vegetables, nuts, and potatoes [54].

Among the strengths of this study, it can be mentioned the use of validated questionnaires, which facilitates the comparison of the results with those obtained in other studies conducted in other parts of the world. Among the weaknesses, we can indicate that it is a cross-sectional study; therefore, it is only possible to establish associations but not to identify causality.

## 5. Strengths and Limitations of the Study

Finally, our study had some limitations that we would like to mention below.

Internet access in the Chilean and Latin American population is not a significant limitation due to the high penetration and use of social networks in households (regardless of socioeconomic level), particularly in Chile, where the majority of responses were concentrated, as 93% of the population has internet connectivity. However, there could still be some limitations to the study, as older adults may face more difficulties and have less familiarity with technology. To mitigate this situation, we considered the support of caregivers and promoted the survey on social media targeted at the disease, patients, and their families.

On the other hand, it is important to note that this is an observational study with a non-probabilistic sampling method, which may present limitations inherent to the methodology. Additionally, as a cross-sectional observational study, we can only speak of associations rather than causality.

Given the extensive length of the survey, which contained 50 questions, it was not possible to inquire about the dosage of medications used for Parkinson’s disease, as we only asked about the medications consumed, and respondents could select all the medications they were taking.

As strengths of this research, it is worth mentioning that it was an exploratory study and serves as a baseline for future studies in our geographical area, as it is the first to evaluate these parameters. The study used only validated surveys (one of which was validated for this research, and the other instruments had been previously validated), which we believe strengthens the internal validity of the study. Additionally, by using validated instruments, our data allows for comparison with other studies.

For the second phase of the study, which will begin shortly, we will incorporate new information, such as the type of medication used for the management of Parkinson’s disease, dosage, and administration timing, as well as a validated dietary survey to determine fiber intake in the target population.

## 6. Conclusions

The use of levodopa was associated with BMI and the SARC-F score. On the other hand, chronic constipation was associated with BMI; thus, we can conclude that nutritional assessment and subsequent follow-up should be conducted in PD patients in order to avoid complications that might be associated with levodopa therapy as well as with the intrinsic effects of the disease. There is a need to conduct clinical trials in order to determine causality and provide recommendations designed to improve the health of people suffering from PD.

Additionally, respondents with sarcopenia, those with less than 5 years with the disease, and individuals with an overweight nutritional status have a higher risk of constipation.

## Figures and Tables

**Table 1:** Overall description of nutritional status and years with Parkinson’s disease.

Variable	No.	Mean ± SD	(Min–Max)
Age	160	60.84 ± 12.13	(21–87)
Weight	158	70.77 ± 16.90	(22–125)
Height	155	1.63 ± 0.09	(1.40–1.87)
BMI	153	26.44 ± 5.69	(12.48–53.21)
SARC-F	90	4.76 ± 2.78	(0–10)
MNA	90	10 ± 3.24	(0–14)
No. of years with Parkinson’s disease	157	9.41 ± 6.97	(0.3–40)

BMI: body mass index; MNA: Mini Nutritional Assessment; SARC-F: questionnaire for detection of sarcopenia.

**Table 2:** Description of the variables constipation, Parkinson’s disease, and nutritional status (MNA).

Variable	Frequency (%)
Constipation Assessment	
Are you constipated? (n = 161)	
Yes	116 (72.05)
No	45 (27.95)
Bristol Scale (n = 161)	
Type 1 and Type 2	64 (39.75)
Type 3 and Type 4	81 (50.31)
Type 5, Type 6, and Type 7	16 (9.94)
Frequency of going to the toilet (n = 161)	
Once or less per week	18 (11.18)
Twice a week	41 (25.47)
Every other day	48 (29.81)
Daily	54 (33.54)
Use of medications for constipation	
Does not use any medication	84(52.50)
Osmotic laxative	28(17.50)
Stimulant laxative	22(13.75)
Natural supplements	26(16.25)
Parkinson’s disease	
Classification of the disease according to Hoehn and Yahr (n = 161)	
Stage 1	49 (30.43)
Stage 2	43 (26.71)
Stage 3	36 (22.36)
Stages 4 and 5	33 (20.50)
Use of levodopa	
No	20 (12.50)
Yes	140 (87.50)
Mini Nutritional Assessment (n = 90)	
Normal	31 (34.44)
Risk of malnutrition	37 (41.11)
Malnutrition	22 (24.44)

SARC-F > 4 points indicative of sarcopenia. Bristol scale: Type 1 and type 2: constipation; type 3 and type 4: normal; type 5, type 6, type 7: precursor to diarrhea and diarrhea. Hoehn and Yahr stages, Stage 1: Early stage, the person has mild symptoms that usually do not interfere with daily activities. Tremors and other motor symptoms occur only on one side of the body. Changes in posture, gait, and facial expression. Stage 2: Symptoms begin to aggravate. Tremor, rigidity, and other motor symptoms affect the body bilaterally. Walking problems and poor posture may be evident. The person can still live on their own, but everyday tasks become more difficult and take longer. Stage 3: Considered an intermediate stage. The person begins to experience balance problems and slowness of movement. Falls are frequent. The person is still fully independent, but symptoms significantly affect activities such as dressing and eating. Stage 4: At this point, there are severe and limiting symptoms. The person may need a walking frame. The person needs assistance with activities of daily living and cannot live on their own. Stage 5: This is the most advanced stage. Leg stiffness can make it impossible to stand or walk. Permanent assistance is required to move autonomously.

**Table 3:** Association between constipation—according to the Bristol scale—and sex, nutritional status, stage of the disease, use of levodopa, and polypharmacy.

Variable	Constipation (Bristol Scale) Freq (%)				*p* Value
	G1	G2	G3	Total	
Sex					
Female	42 (39.25)	52 (48.60)	13 (12.15)	107 (100)	*p* = 0.411
Male	22 (40.74)	29 (53.70)	3 (5.56)	54 (100)	
Nutritional status (BMI)					
Underweight	18 (60.00)	10 (33.33)	2 (6.67)	30 (100)	*p* = 0.022
Normal weight	22 (40.00)	30 (54.55)	3 (5.45)	55 (100)	
Overweight	7 (21.88)	22 (68.75)	3 (9.38)	32 (100)	
Obesity	14 (38.89)	15 (41.67)	7 (19.44)	36 (100)	
Nutritional Status—Mini Nutritional Assessment (MNA)					
Normal	11 (35.48)	17 (54.84)	3 (9.68)	31 (100)	*p* = 0.825
Risk of malnutrition	17 (45.95)	16 (43.24)	4 (10.81)	37 (100)	
Malnutrition	10 (45.45)	11 (50)	1 (4.55)	22 (100)	
Stage of the disease (Hoehn and Yahr)					
Stage 1	14 (28.57)	29 (59.18)	6 (12.24)	49 (100)	*p* = 0.296
Stage 2	15 (34.88)	24 (55.81)	4 (9.30)	43 (100)	
Stage 3	17 (47.22)	15 (41.67)	4 (11.11)	36 (100)	
Stage 4 and Stage 5	18 (54.55)	13 (39.39)	2 (6.06)	33 (100)	
Use of levodopa					
Yes	56 (40.00)	70 (50.00)	14 (10.00)	140 (100)	*p* = 0.939
No	7 (35.00)	11 (55.00)	2 (10.00)	20 (100)	
Polypharmacy (3 or more medications)					
YES	10 (40.00)	14 (56.00)	1 (4.00)	25 (100)	*p* = 0.628
NO	54 (39.71)	67 (49.26)	15 (11.03)	136 (100)	

Freq: frequency; G1: individuals who reported types 1 and 2 of the Bristol scale (constipation); G2: individuals who reported types 3 and 4 of the Bristol scale (normal); G3: individuals who reported types 5, 6, and 7 of the (loose stools). Classification of nutritional status: underweight (BMI < 22); normal weight (BMI: 22–27); overweight (BMI: >27–32); obesity (BMI > 32). Hoehn and Yahr stages: Stage 1: Early stage, the person has mild symptoms that usually do not interfere with daily activities. Tremors and other motor symptoms occur only on one side of the body. Changes in posture, gait, and facial expression. Stage 2: Symptoms begin to aggravate. Tremor, rigidity, and other motor symptoms affect the body bilaterally. Walking problems and poor posture may be evident. The person can still live on their own, but everyday tasks become more difficult and take longer. Stage 3: Considered an intermediate stage. The person begins to experience balance problems and slowness of movement. Falls are frequent. The person is still fully independent, but symptoms significantly affect activities such as dressing and eating. Stage 4: At this point, there are severe and limiting symptoms. The person may need a walking frame. The person needs assistance with activities of daily living and cannot live on their own. Stage 5: This is the most advanced stage. Leg stiffness can make it impossible to stand or walk. Permanent assistance is required to move autonomously. Fisher’s test, a *p*-value of <0.05 was considered to indicate a significant association.

**Table 4:** Association between use of levodopa and nutritional status, sex, stage of the disease, and polypharmacy.

Variable	Use of Levodopa Freq (%)			*p* Value
	YES	NO	Total	
Nutritional status (BMI)				
Underweight	26 (86.67)	4 (13.33)	30 (100)	*p* = 0.049
Normal weight	49 (90.74)	5 (9.26)	54 (100)	
Overweight	23 (71.88)	9 (28.13)	32 (100)	
Obesity	34 (94.44)	2 (5.56)	36 (100)	
Nutritional Status—Mini Nutritional Assessment (MNA)				
Normal	26 (83.87)	5 (16.13)	31 (100)	*p* = 0.470
Risk of malnutrition	32 (86.49)	5 (13.51)	37 (100)	
Malnutrition	21 (95.45)	1 (4.55)	22 (100)	
Stage of the disease (Hoehn and Yahr)				
Stage 1	43 (87.76)	6 (12.24)	49 (100)	*p* = 0.798
Stage 2	39 (90.70)	4 (9.30)	43 (100)	
Stage 3	30 (83.33)	6 (16.67)	36 (100)	
Stage 4 and Stage 5	28 (87.50)	4 (12.50)	32 (100)	
Sex				
Female	95 (89.62)	11 (10.38)	106 (100)	*p* = 0.255
Male	45 (83.33)	9 (16.67)	54 (100)	
Polypharmacy (3 or more medications)				
YES	24 (100)	0 (0)	24 (100)	*p* = 0.046
NO	116 (85.29)	20 (14.71)	136 (100)	

Freq: frequency; Classification of nutritional status: underweight (BMI < 22); normal weight (BMI: 22–27); overweight (BMI: >27–32); obesity (BMI > 32). Hoehn and Yahr stages: Stage 1: Early stage, the person has mild symptoms that usually do not interfere with daily activities. Tremors and other motor symptoms occur only on one side of the body. Changes in posture, gait, and facial expression. Stage 2: Symptoms begin to aggravate. Tremor, rigidity, and other motor symptoms affect the body bilaterally. Walking problems and poor posture may be evident. The person can still live on their own, but everyday tasks become more difficult and take longer. Stage 3: Considered an intermediate stage. The person begins to experience balance problems and slowness of movement. Falls are frequent. The person is still fully independent, but symptoms significantly affect activities such as dressing and eating. Stage 4: At this point, there are severe and limiting symptoms. The person may need a walking frame. The person needs assistance with activities of daily living and cannot live on their own. Stage 5: This is the most advanced stage. Leg stiffness can make it impossible to stand or walk. Permanent assistance is required to move autonomously. Fisher’s test, a *p*-value of <0.05 was considered to indicate a significant association.

**Table 5:** Comparison between constipation (according to the Bristol scale) and the variables sarcopenia, age, and time suffering from Parkinson’s disease.

Variable	Constipation Mean ± SD				
	G1	G2	G3	Total	*p* Value
Age	62.62 ± 12.99	59.51 ± 11.28	60.37 ± 12.51	60.84 ± 12.13	*p* = 0.3080
SARC-F score	5.39 ± 2.54	4.23 ± 2.84	4.63 ± 3.25	4.76 ± 2.78	*p* = 0.1650
No. of years with Parkinson’s disease	11.76 ± 7.77	8 ± 6.30	8.29 ± 4.62	9.47 ± 6.98	*p* = 0.0047

SARC-F score: >4 points is indicative of sarcopenia; G1: individuals who reported types 1 and 2 of the Bristol scale (constipation); G2: individuals who reported types 3 and 4 of the Bristol scale (normal); G3: individuals who reported types 5, 6, and 7 of the Bristol scale (loose stools). ANOVA test, multiple comparisons according to Bonferroni *p* < 0.05.

**Table 6:** Comparison between use of levodopa and the variables sarcopenia, age, and time suffering from Parkinson’s disease.

Variable	Use of Levodopa				*p* Value
	YES		NO		
	Mean ± SD	95% CI	Mean ± SD	95% CI	
SARC-F score	4.97 ± 2.63	(4.39–5.56)	3.18 ± 3.46	(0.86–5.51)	*p* = 0.0446
Age (years)	61.17 ± 11.97	(59.17–63.18)	59.1 ± 13.37	(52.83–65.36)	*p* = 0.4752
Time with Parkinson’s disease (years)	9.48 ± 6.75	(8.33–10.62)	9.13 ± 8.73	(4.91–13.34	*p* = 0.8385

SARC-F score: >4 points is indicative of sarcopenia. ANOVA test, multiple comparisons according to Bonferroni *p* < 0.05.

**Table 7:** Regression for the probability of constipation with BMI.

	Odd Ratio	95% CI (Min–Max)	*p* Value
SARC-F score	2.902	(1.070–7.866)	0.036
Time with Parkinson’s disease			
<5 years	3.711	(1.171–11.760)	0.026
5–10 years	1.617	(0.646–4.049)	0.304
Levodopa consumption	1.338	(0.406–4.403)	0.631
Stage of the disease (Hoehn and Yahr)			
Stages 1 and 2	1.334	(0.403–4.411)	0.636
Stage 3	1.752	(0.441–6.956)	0.425
Stages 4 and 5	1.332	(0.305–5.799)	0.702
Nutritional status according to BMI			
Normal	1.832	(0.593–5.654)	0.292
Overweight	4.450	(1.172–16.886)	0.028
Obesity	2.062	(0.602–7.059)	0.249
Use of medications for constipation			
Osmotic laxative	0.575	(0.208–1.588)	0.286
Stimulant laxative	0.341	(0.105–1.110)	0.074
Natural supplements	1.025	(0.323–3.245)	0.966

Hosmer–Lemeshow test (*p* = 0.5409). A *p*-value < 0.005 is considered significant.

**Table 8:** Logistic regression for the probability of constipation with MNA score.

	Odd Ratio	95% CI (Min–Max)	*p* Value
SARC-F score	2.784	(1.085–7.143)	0.033
Time with Parkinson’s disease			
<5 years	3.461	(1.207–9.922)	0.021
5–10 years	1.710	(0.704–4.153)	0.236
Levodopa consumption	0.947	(0.312–2.869)	0.924
Stage of the disease (Hoehn and Yahr)			
Stages 1 and 2	1.645	(0.547 -4.940)	0.375
Stage 3	1.870	(0.507–6.892)	0.347
Stages 4 and 5	1.618	(0.388–6.737)	0.508
Nutritional status according to BMI			
Normal	1.515	(0.518–4.429)	0.447
Risk of malnutrition	1.270	(0.511–3.160)	0.606
Use of medications for constipation			
Osmotic laxative	0.612	(0.228–1.638)	0.329
Stimulant laxative	0.412	(0.142–1.190)	0.101
Natural supplements	1.251	(0.414–3.781)	0.691

Homer–Lemeshow Test (*p* = 0.5021). A *p*-value < 0.005 is considered significant.

## Data Availability

The original contributions presented in the study are included in the article. Further inquiries can be directed to the corresponding author.
